# Preparation, Characterization, and Evaluation of Cisplatin-Loaded Polybutylcyanoacrylate Nanoparticles with Improved In Vitro and In Vivo Anticancer Activities

**DOI:** 10.3390/ph13030044

**Published:** 2020-03-11

**Authors:** Mohsen Ghaferi, Samar Amari, Bhalchandra Vivek Mohrir, Aun Raza, Hasan Ebrahimi Shahmabadi, Seyed Ebrahim Alavi

**Affiliations:** 1 Department of Pilot Nanobiotechnology, Pasteur Institute of Iran, Tehran 1316943551, Iran; mn.ghaferi@gmail.com; 2 Department of Chemical Engineering, Shahrood Branch, Islamic Azad University, Shahrood 36155-163, Iran; 3 School of Mechanical, Medical and Process Engineering, Queensland University of Technology (QUT), Brisbane, Queensland 4000, Australia; samar.amari@hdr.qut.edu.au; 4 School of Pharmacy, The University of Queensland, Woolloongabba 4102, Australia; b.mohrir@uqconnect.edu.au (B.V.M.); aun.raza@uq.edu.au (A.R.); 5 Department of Microbiology, School of Medicine, Rafsanjan University of Medical Sciences, Rafsanjan 7717933777, Iran

**Keywords:** cisplatin, cytotoxicity, drug delivery, kidney cancer, PBCA nanoparticles

## Abstract

This study aimed to evaluate the therapeutic efficacy of the cisplatin encapsulated into polybutylcyanoacrylate (PBCA) nanoparticles for the treatment of kidney cancer. The nanoformulation was successfully developed using the miniemulsion polymerization method and characterized in terms of size, size distribution, drug loading and encapsulation efficiencies, drug release behavior, in vitro cytotoxicity effects, in vivo toxicity, and therapeutic effects. Cisplatin-loaded PBCA nanoparticles were confirmed to be in nanoscale with the drug entrapment efficiency of 23% and controlled drug release profile, in which only 9% of the loaded drug was released after 48 h. The nanoparticles caused an increase in the cytotoxicity effects of cisplatin against renal cell adenocarcinoma cells (ACHN) (2.3-fold) and considerably decreased blood urea nitrogen and creatinine concentrations when compared to the standard cisplatin (1.6-fold and 1.5-fold, respectively). The nanoformulation also caused an increase in the therapeutic effects of cisplatin by 1.8-fold, in which a reduction in the mean tumor size was seen (3.5 mm vs. 6.5 mm) when compared to the standard cisplatin receiver rats. Overall, cisplatin-loaded PBCA nanoparticles can be considered as a promising drug candidate for the treatment of kidney cancer due to its potency to reduce the side effects of cisplatin and its toxicity and therapeutic effects on cancer-bearing Wistar rats.

## 1. Introduction

Renal cell carcinoma (RCC; also known as renal adenocarcinoma), with increasing incidence, causes 90% of renal malignancies [1] and is responsible to 2.2% of all malignancies [2]. RCC is a highly metastasis malignancy [3] that 25–30% of RCC patients are diagnosed with metastasis [1]. Furthermore, it is one of the most lethal cancers with a five-year survival rate of 15–25% [4]. One study showed that nearly 270,000 new cases are diagnosed with RCC, which causes approximately 116,000 deaths worldwide [5]. Currently, chemotherapy is considered as a main approach for the treatment of kidney cancer [6], and cisplatin is one the chemotherapeutic agent used for the treatment of RCC [3].

Cisplatin plays a vital role in cancer treatment and has been widely used to treat various types of cancers, including ovarian, cervical, head and neck, and non-small-cell lung cancer for years [7]. It forms a cisplatin-DNA adduct, which causes cell death through the cell apoptotic process [8]. However, cisplatin has several severe side effects, including gastrotoxicity, ototoxicity, allergic reactions, and myelosuppression that limit its clinical use [9]. Today, nanoparticles are considered as a promising approach for improving the pharmacokinetics of various types of drugs [10,11].

Nanoparticles have been widely investigated as drug delivery systems to increase the therapeutic efficacy of drugs [12,13,14,15,16] and reduce their side effects [17,18]. Nanoparticles have a high surface area to volume ratio; therefore, they could encapsulate drugs in high concentrations and increase drug delivery to cancerous cells [19]. They are also considered as excellent drug delivery systems, due to isolating the chemotherapeutic compounds from the systemic environment and targeted accumulation of the compounds in solid tumors with leaky vasculature and impaired lymphatics, resulting in improved cellular uptake [20]. In addition, nanoparticles are able to increase the water solubility of small insoluble molecules, leading to improving their solubility and bioavailability [21]. Polybutylcyanoacrylate (PBCA) nanoparticles are polymeric carriers and have been broadly used for drug delivery purposes. They are biodegradable particles and potent to alter the biological distribution of therapeutic agents in the human body. Apart from this, PBCA nanoparticles take advantage of easy preparation and purification processes and being able to overcome multidrug resistance [22,23]. These features make these particles as a suitable candidate for drug delivery. The present study aimed to use this carrier for cisplatin delivery to RCC. For this purpose, the potency of PBCA as the cisplatin carrier was evaluated in vitro and in vivo environments for the treatment of kidney cancer. In this regard, the in vitro release behavior, cytotoxicity, in vivo toxicity, and therapeutic effects of the formulation were studied using the dialysis membrane method, 3-[4,5-dimethylthiazole-2-yl]-2,5-diphenyltetrazolium bromide (MTT) assay, and histopathological studies. Further analyses, including blood urea nitrogen (BUN) and creatinine concentrations, and their tumor size were also investigated to evaluate the toxicity of the nanoformulation.

## 2. Results and Discussion

### 2.1. Characterization of Nanoparticles

**Figure 1:**
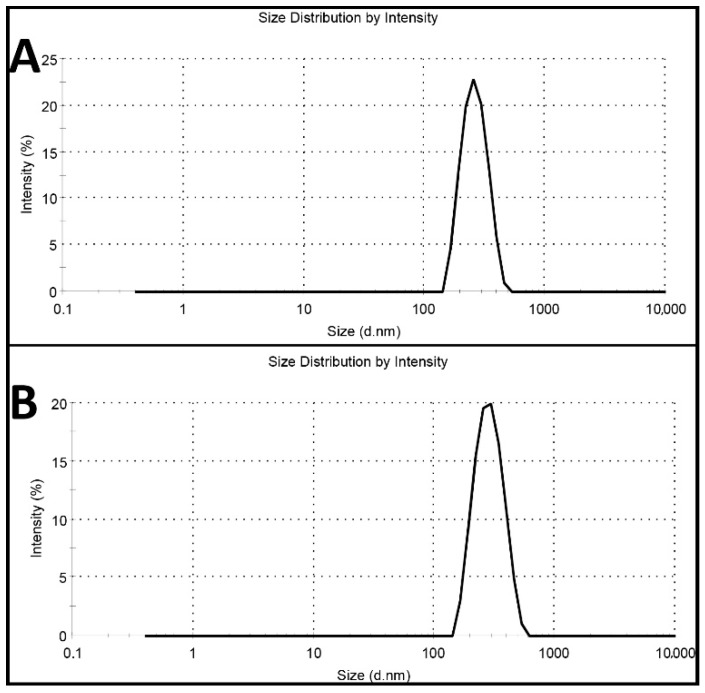
Intensity profile of (**A**) blank polybutylcyanoacrylate (PBCA) nanoparticles and (**B**) cisplatin-loaded PBCA nanoparticles.

Cisplatin-loaded PBCA nanoparticles were synthesized using miniemulsion polymerization method. In this method, dextran 70 kDa was used as a stabilizer to prevent macroaggregation of the nanoparticles [24]. Among various kinds of polyalkylcyanoacrylate nanoparticles, such as polymethyl, polyethyl, polypropyl, and polybutyl, PBCA has been commonly used for drug delivery, owning to its ability to interact with the different types of drugs [25]. Also, in cancer therapy, various studies have shown that PBCA nanoparticles are excellent carrier colloids for chemotherapeutic agents as they enhance the therapeutic effects of chemotherapeutics and reduce their side effects [9,26,27,28,29]. In the current study, honey and olive oil were used for stabilizing the nanoparticles. Researchers, in various studies, have also used these compounds to stabilize nanoparticles [30,31,32,33]. Moreover, honey and olive oil were used because of their surfactant activity and anticancer properties [23]. Surfactants inhibit nanoparticles’ aggregation through creating a physical barrier (a shell surrounding the particles) outside the particles [34,35]. In this study, the polymerization of BCA monomer was confirmed by the color change of the reaction medium from colorless to milky [22]. To confirm the monodispersity of the nanoparticles, dynamic light scattering (DLS) method was used, and the results showed that the nanoparticles were monodispersed (Figure 1).

**Figure 2:**
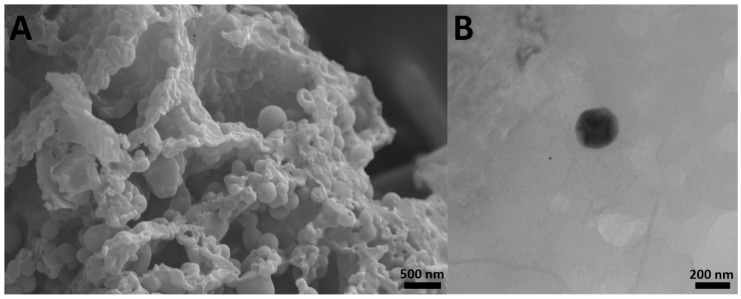
(**A**) scanning electron microscopy SEM and (**B**) transmission electron microscopy TEM images of cisplatin-loaded PBCA nanoparticles prepared using miniemulsion polymerization method (×15,000).

The results also showed that cisplatin-loaded PBCA nanoparticles were formed in nanoscale size and with a negative zeta potential (Figure 1, Table 1). The size of nanoparticles is a critical factor in investigating the efficacy of loaded chemotherapeutics, in which smaller nanoparticles have higher cellular uptake and cellular transfection efficiency, resulting in a higher intracellular concentration of the loaded chemotherapeutics [36]. Particles with the size below 300 nm can efficiently enter target cells to exert the pharmaceutical function [37]. The size of cisplatin-loaded PBCA nanoparticles prepared in the present study was 274 ± 6.7 nm; thus, they can effectively enter the target cells. The zeta potential of the blank and cisplatin-loaded nanoparticles was found to be −7 ± 0.30 mV and −9 ± 0.42 mV, respectively. The colloidal stability of positive or negative charged nanoparticles is generally preserved in aqueous solutions with low ionic strength, due to the repulsive forces [38]. However, cisplatin-loaded PBCA nanoparticles showed more positive zeta potential compared to the PBCA nanoparticles. This is due to cisplatin has a positive charge [39], and it was loaded into the nanoparticles. Drug loading efficiency is a critical factor in polymeric carriers, for instance, low drug loading is responsible for both poor therapeutic efficacy and drug release profiles [40], resulting in the insufficient efficiency of drug delivery systems [41]. Therefore, improving drug loading efficiency is critical to achieving sufficient therapeutic activity [42]. This study showed that despite the low water solubility of cisplatin [43], drug loading efficiency of 23% was achieved. This confirmed that the miniemulsion polymerization method was efficient enough to prepare the nanoparticles. The shape and morphology of nanoparticles also play a critical role in the potency of nanoparticles as a drug delivery system, affecting the pharmacokinetics of the particles in the body [44]. In the current study, the morphology of the nanoparticles was precisely studied using scanning electron microscopy (SEM) and transmission electron microscopy (TEM) (Figure 2). The SEM results showed that spherical monodisperse nanoparticles with the smooth surface and without surface fractures or pitting were formed (Figure 2A). The results of TEM confirmed the formation of spherical monodispersed cisplatin-loaded PBCA nanoparticles as solid nanoparticles (Figure 2B).

**Figure 3:**
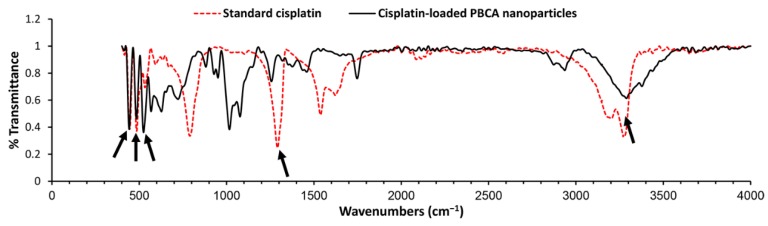
Fourier-transform infrared (FTIR) spectrum of the standard cisplatin and cisplatin-loaded PBCA nanoparticles. Arrows show the position of chemical bonds of cisplatin [26].

To evaluate the chemical structure of cisplatin and determine if it preserves its chemical structure, cisplatin-loaded PBCA nanoparticles were investigated using the Fourier-transform infrared (FTIR) spectrum. Changes in chemical structures of therapeutic compounds can change their biological activity [45]. In other words, chemical structure determines the biological properties of substances [46]. According to the results of the FTIR spectrum (Figure 3), the symmetric amine bending mode at 1290 cm^−1^ and the characteristic amine stretching mode at 3280 and 3200 cm^−1^ frequency regions appeared. According to these findings, which correspond to the FTIR spectrum of cisplatin [26,47], cisplatin remained intact in PBCA nanoparticles, i.e., cisplatin was loaded into the nanoparticles physically without forming any chemical bonds.

### 2.2. Drug Release Study

**Figure 4:**
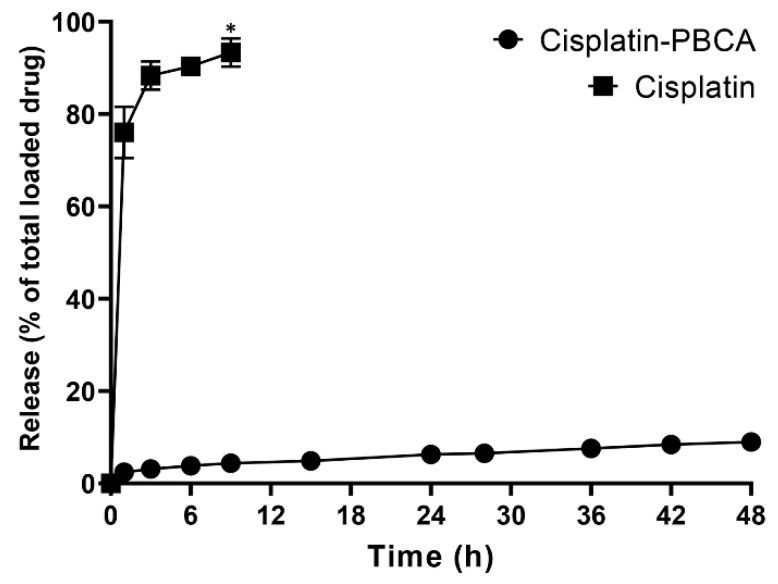
Release pattern of cisplatin from the standard cisplatin solution and PBCA nanoformulation. The results were expressed as the values ±5% from three independent experiments. Statistical analyses were performed using one-way ANOVA, F-test with *p < 0.05. The data were expressed as mean ± SD (n = 3).

Drug delivery systems with a controlled release rate have substantial advantages compared to the conventional dosage forms. These systems (i) considerably decrease the dosing frequency and improve patient compliance, (ii) minimize the in vivo fluctuation of drug concentrations and maintain the drug concentrations within the desired range, (iii) can be used for localized drug delivery, and (iv) decrease the drug side effects [48]. In the current study, 76% of the cisplatin was released from the cisplatin solution during the first one hour, while the cumulative drug release from the nanoparticles was slow. A burst drug release occurred from cisplatin-loaded PBCA nanoparticles in the first hour of the study, in which 28% of the cisplatin was released from the nanoformulation (Figure 4). The burst drug release from nanoparticles was also reported by other researchers [49,50], and could result from the release of the adsorbed drug onto the nanoparticles or weakly bound between the drug and the surface of nanoparticles [51]. Tian et al. [49] used PBCA nanoparticles as a drug carrier to deliver temozolomide into the brain, and they observed a 30% drug release in the first hour. This was also reported by Zhang et al. [50] that 33% of the loaded insulin was released from PBCA nanoparticles in the first one hour. In the present study, a slow-controlled drug release from the nanoparticles was observed, in which only 9% of the loaded drug was released after 48 h.

### 2.3. In Vitro Cytotoxicity Effects

**Figure 5:**
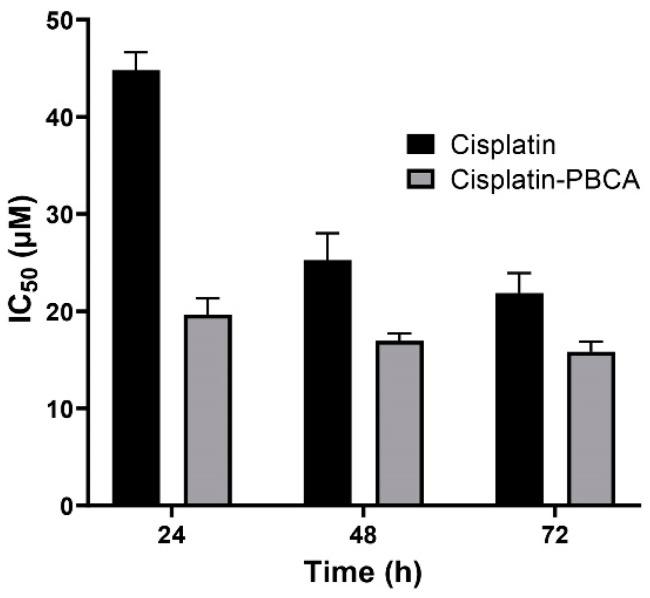
Cytotoxicity effects (IC_50_) of cisplatin-loaded PBCA nanoparticles (19.2 ± 0.41, 17 ± 0.9 and 15 ± 0.27 µM after 24-, 48- and 72-h incubation, respectively) compared to the standard drug (44.4 ± 1.9, 26 ± 0.83, and 20.3 ± 0.22 µM after 24-, 48-, and 72-h incubation, respectively) against human renal adenocarcinoma (ACHN). The data were expressed as mean ± SD (n = 3).

**Figure 6:**
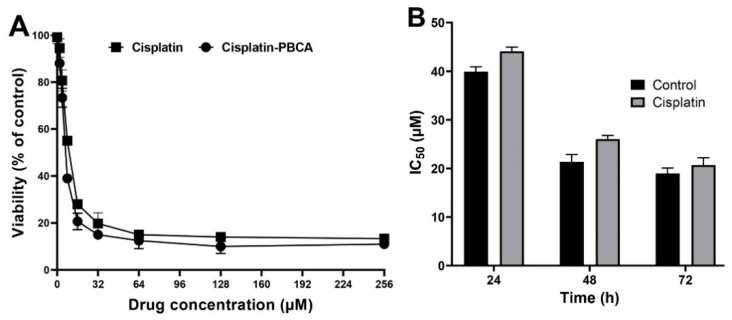
(**A**) Viability effects (% of control) of cisplatin-loaded PBCA nanoparticles compared to the standard drug (cisplatin) on human renal adenocarcinoma (ACHN) cells after 24-h incubation; and (**B**) cytotoxicity effects (IC_50_) of the control (the combination of the free drug with blank PBCA nanoparticles) and cisplatin against ACHN cells (39.9 ± 1.0, 21.4 ± 1.5, and 19.0 ± 1.1 µM after 24-, 48- and 72-h incubation, respectively). The data were expressed as mean ± SD (n = 3).

**Figure 7:**
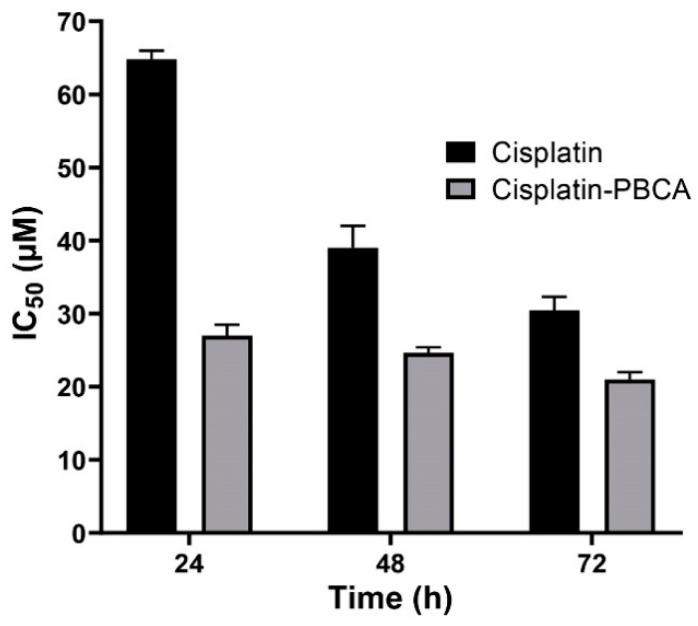
Cytotoxicity effects (IC_50_) of cisplatin-loaded PBCA nanoparticles (27 ± 1.32, 24.6 ± 1.2, and 21 ± 1.07 µM after 24-, 48-, and 72-h incubation, respectively) compared to the standard drug (64.8 ± 3.22, 39 ± 1.91, and 30.45 ± 1.51 µM after 24-, 48-, and 72-h incubation, respectively) against BHK-21. The data were expressed as mean ± SD (n = 3).

**Figure 8:**
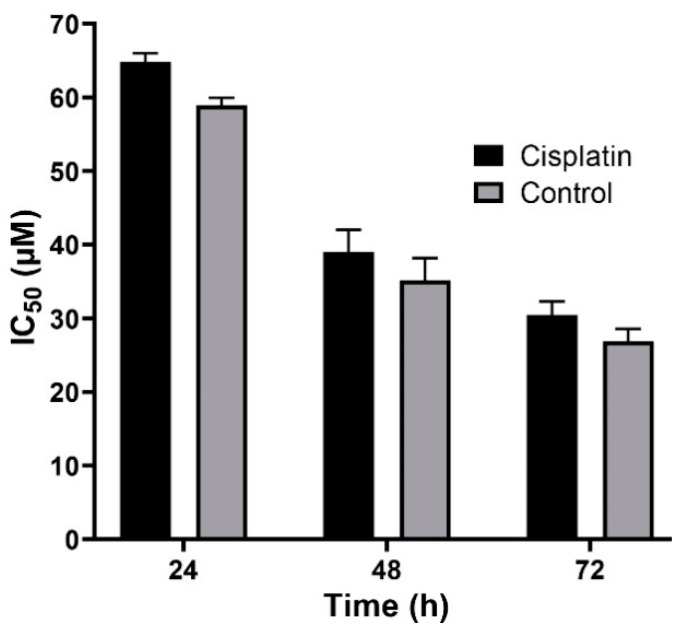
Cytotoxicity effects (IC_50_) of the control (the combination of the free drug with blank PBCA nanoparticles) and cisplatin against BHK-21 cells (39.9 ± 1.0, 21.4 ± 1.5, and 19.0 ± 1.1 µM after 24-, 48-, and 72-h incubation, respectively). The data were expressed as mean ± SD (n = 3).

Nanoparticles play an important role in the treatment of cancers as they can (i) increase drug concentration in cancerous cells, due to a prolonged circulation which helps nanoparticles to meet their targets, and (ii) prevent drug toxicity in normal cells, due to targeting capacity in an in vivo environment [52,53]. Various studies showed that drug encapsulation into nanocarriers increased their cytotoxicity effects on the cancerous cells [13,15,16]. In the present study, the non-toxic concentration of PBCA nanoparticles was determined to be 25 and 38 µg/mL for ACHN and BHK-21 cells, respectively, and these concentrations were used for the cytotoxicity study. The results of this study showed that cisplatin-loaded PBCA nanoparticles significantly increased the cytotoxicity of cisplatin (*p* < 0.05) against both cell lines. This finding was more prominent in the 24-h evaluation, where the cytotoxicity of the nanoformulation was increased by 2.3-fold and 2.4-fold compared to that of the standard cisplatin toward ACHN and BHK-21 cells, respectively (Figure 5, Figure 6A, and Figure 7). Also, the cytotoxicity of cisplatin-loaded PBCA nanoparticles did not significantly change in three different incubation times, in which the cytotoxicity (IC_50_) of the nanoformulation against ACHN cells was 19.2, 17, and 15 µM for 24, 48, and 72 h incubation time, respectively, and the IC50 of the nanoformulation against BHK-21 cells was 27, 24.65, and 21 µM, for 24, 48, and 72 h incubation time, respectively. This indicated that cisplatin was released from the nanoparticles in a slow-controlled release manner [54,55] as only 9% of the loaded drug was released from the nanoparticles after 48 h incubation. The enhanced cytotoxicity of cisplatin-loaded PBCA nanoparticles compared to free cisplatin resulted from slow-controlled drug release from the particles [11]. It is also due to the particles enter the cells more efficiently [56]. Also, as the results showed, the non-tumoral kidney cells (BHK-21) were less sensitive compared to the tumoral ones (human renal adenocarcinoma—ACHN) toward cisplatin and cisplatin-loaded PBCA nanoparticles, confirming that the cytotoxicity effects of cisplatin and cisplatin-loaded PBCA nanoparticles were selective against tumoral cells rather than non-tumoral ones. In addition, the cytotoxicity effects of cisplatin in combination with blank PBCA nanoparticles, as a control, against both ACHN and BHK-21 cells were evaluated, and the results demonstrated that its cytotoxicity was increased compared to the free drug against both cell lines (Figure 6B and Figure 8). This finding might result from the cisplatin adsorption onto the nanoparticles and increase in the cisplatin transport across the cell membrane. Also, the cytotoxicity of the control was significantly lower compared to that of cisplatin-loaded PBCA nanoparticles against both cell lines (*p* < 0.05).

### 2.4. In Vitro Stability

**Figure 9:**
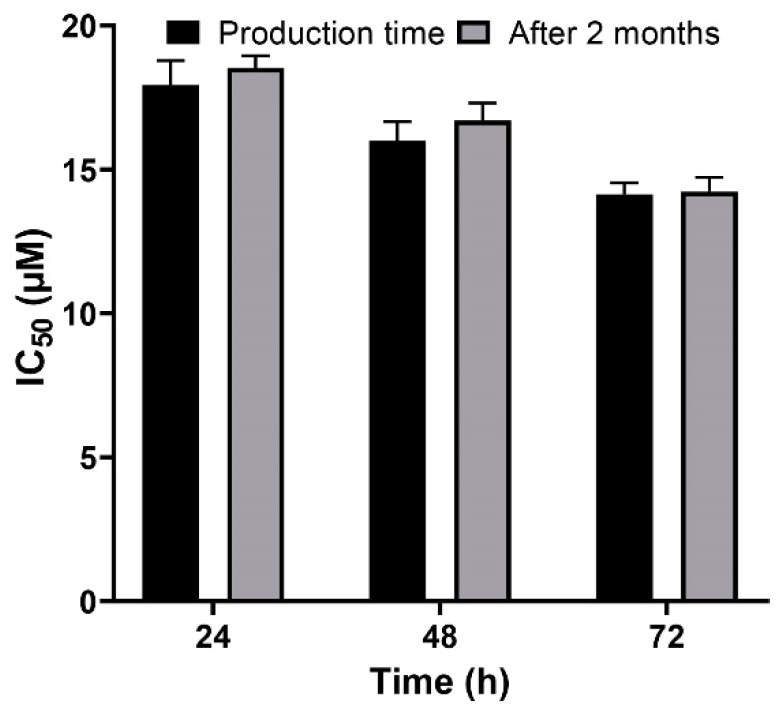
Evaluation of the cytotoxicity effects of cisplatin-loaded PBCA nanoparticles in the production time compared to two months after the synthesis. The data were expressed as mean ± SD (n = 3).

A versatile drug delivery carrier should demonstrate carrier and encapsulation stability, which restrain premature drug release prior to approaching the target site [57], and for this reason, stability is considered as a key factor for the development of formulations. PBCA nanoparticles have been known as stable carriers due to the rigidity of their matrix/shell [58]. In the present study, the results showed that the cytotoxicity effect of cisplatin-loaded PBCA nanoparticles, two months after the synthesis, was approximately similar to the results obtained in the first day of the synthesis (Figure 9), indicating the sufficient stability of the nanoformulation and, consequently, its ability to maintain the anticancer effects of cisplatin.

### 2.5. In Vivo Toxicity and Therapeutic Effects

In the present study, renal cancer was successfully developed using FeNTA solution as it results in a high incidence of kidney cancer in mice [59]. The results of histopathological studies showed that 72% of rats received FeNTA developed kidney cancer. From this value, 75% showed bilateral kidney tumors, while in the remaining animals, unilateral tumors were observed. Also, it was found FeNTA was toxic; however, it caused animals death at the high doses (9 mg Fe/kg), in which eight rats (14% of the total number) died. The results also showed that FeNTA significantly increased BUN and creatinine by 13-fold and 14-fold, respectively, compared to the healthy control group (Table 2), indicating renal failure. These results confirmed the toxicity effects of FeNTA.

**Figure 10:**
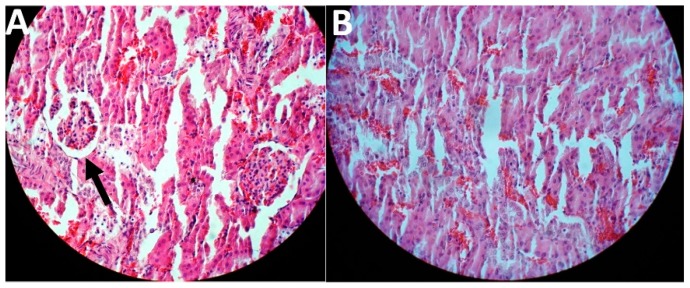
Histological evaluation of kidney tissue in kidney cancer-bearing rats received (**A**) the standard cisplatin and (**B**) cisplatin-loaded PBCA nanoformulation. The arrow shows ATN.

In this study, the treatment was initiated two weeks after the last FeNTA injection. The results of toxicity evaluation showed that cisplatin-loaded PBCA nanoparticles were more potent in decreasing the blood concentration of BUN and creatinine, compared to the standard drug by 1.6-fold and 1.5-fold, respectively, and to the PBS group by 1.9-fold and 2.1-fold, respectively (Table 2). In addition, cisplatin-loaded PBCA nanoparticles caused a significant reduction in the tumor size by 1.8-fold compared to that of the standard cisplatin receiver group, and by 2.6-fold compared to the PBS receiver group (the mean tumor size was 2.5, 3.5, and 6.5 mm in cisplatin-loaded PBCA nanoparticles, standard cisplatin, and PBS receiver groups, respectively). The results of histopathological studies also showed that cisplatin-loaded PBCA nanoparticles significantly decreased the kidney toxicity of cisplatin, in which lower acute tubular necrosis (ATN) was observed in cisplatin-loaded PBCA receiver group compared to the free cisplatin receiver group (Figure 10).

## 3. Materials and Methods

### 3.1. Materials

BCA monomer and dextran 70 kDa were purchased from Evobond^®^Tong Shen Enterprise Co., Ltd. (Kaohsiung, Taiwan) and Zhejiang Chemicals Import and Export Corporation (Hangzhou, China), respectively. Hydrogen chloride, MTT, sodium dodecyl sulfate (SDS), phosphate buffer saline (PBS), sodium hydroxide, hematoxylin and eosin (H&E), hexachloroplatinic acid (H_2_PtCl_6_), dialysis bag (cut-off of 10 KDa), ferric nitrate, nitrilotriacetic disodium salt, sodium bicarbonate, N-diethylnitrosamine (DEN), and cisplatin were purchased from Sigma-Aldrich (St. Louis, MO, USA). Roswell Park Memorial Institute (RPMI)-1640 medium, penicillin/streptomycin antibiotics, and fetal bovine serum (FBS) were purchased from Gibco (Carlsbad, CA, USA). Male Wistar rats (two weeks old, 70–80 g) and human renal adenocarcinoma (ACHN) and non-tumor hamster kidney cells (BHK-21) were prepared from Pasteur Institute of Iran, Tehran. Polyethylene glycol 400 (PEG400) was obtained from Kimiagaran Emrooz Chemical Ind. (Tehran, Iran). All other materials were of analytical grade. Deionized water was used throughout the study. Honey was characterized in terms of pH and moisture content [60] using pH meter and refractometer, respectively. Also, olive oil was characterized according to the previous study [61], in terms of acidity (defined as the required KOH (mg) to neutralize the free fatty acids in 1.0 g of oil), peroxide (as the mEq of O_2_ in the form of peroxide per kg of oil), and iodine (which determines the quantity of unsaturated fatty acids in oil in centigrams (cg) of I_2_ absorbed per gram of sample) rates. Each analysis was performed three times.

### 3.2. Preparation of Cisplatin-Loaded PBCA Nanoparticles

PBCA nanoparticles were prepared using miniemulsion polymerization technique [26]. For this purpose, 250 μL of BCA monomer was added dropwise into a mixture of HCl (125 μL, 0.01 N), olive oil (62.5 μL, peroxide value 3.7 mEq O_2_ kg^−1^ of oil, acidity value 2.8% oleic acid, and iodine value 86.4 cg I2 g^−1^; Faraz Rahbar Saba Co. Tehran, Iran), honey (125 mg, pH: 4.1, moisture content of 25%; Honey Company Naseri, Shiraz, Iran), and PEG400 (125 μL). Next, 187 mg of dextran and 75 mg of cisplatin were added to the resultant and mixed. Then, 25 mL of deionized water was added to the reaction medium, and the mixture was stirred (300 RPM, 10 min, room temperature) to obtain the pre-emulsion suspension. The pre-emulsion suspension was sonicated (Bandelin Sonopuls HD 2070, Berlin, Germany) for 6 × 60 s pulses and 30 s cooling intervals between the pulses. The sonicated pre-emulsion suspension was stirred (150 RPM, 3.5 h) and then neutralized using 0.1 N NaOH to ensure the total consumption of BCA monomers. The blank nanoparticles were also synthesized with the above-mentioned method except that cisplatin was not added to the medium.

### 3.3. Characterization of Nanoparticles

Cisplatin-loaded PBCA nanoparticles were characterized in terms of size, size distribution, zeta potential, drug loading and encapsulation efficiencies, morphology, and chemical structure.

#### 3.3.1. Size, Size Distribution, and Zeta Potential

Here, 50 µL of the nanoparticle suspension (with and without cisplatin) was diluted to 1 mL in deionized water, and its absorbance was read at 620 nm using Malvern Zetasizer Nano ZS (ZEN 3600) (Malvern Instruments Ltd., Malvern, Worcestershire, UK).

#### 3.3.2. Drug Loading and Encapsulation Efficiencies

Drug encapsulation and loading efficiencies were measured using atomic absorption spectrometry (AAS) method, and H2PtCl6 was considered as the standard. For this purpose, the suspension of cisplatin-loaded PBCA nanoparticles was centrifuged (20,000 RPM, 4 °C, 30 min), and the supernatant was obtained. The cisplatin concentration in the supernatant was then measured using ContrAA 700 (Analytik Jena, Jena, Germany) high-resolution continuum source atomic absorption spectrometer. The drug encapsulation and loading efficiencies were measured using Equation (1) and Equation (2).

(1) $$\mathrm{Encapsulation}\;\mathrm{effeciency}\;(\%)=\frac{\mathrm{Initial}\;\mathrm{drug}\;\mathrm{concentration}\;(\mathrm{mg})-\mathrm{drug}\;\mathrm{concentration}\;\mathrm{in}\;\mathrm{supernatant}\;(\mathrm{mg})}{\mathrm{Initial}\;\mathrm{drug}\;\mathrm{concentration}\;(\mathrm{mg})}\times 100$$

(2) $$\mathrm{Loading}\;\mathrm{effeciency}\;(\%)=\frac{\mathrm{Amount}\;\mathrm{of}\;\mathrm{loaded}\;\mathrm{drug}\;\mathrm{in}\;\mathrm{nanoparticles}\;(\mathrm{mg})}{\mathrm{Weight}\;\mathrm{of}\;\mathrm{nanoparticles}\;(\mathrm{mg})}\times 100$$

#### 3.3.3. Morphology

The morphology of the nanoparticles was assessed using SEM and TEM instruments. For this purpose, the suspension of cisplatin-loaded PBCA nanoparticles was lyophilized (Edwards High Vacuum, Manor Royal, Crawley, Sussex, UK), coated with gold, and observed using a KYKY EM-3200 scanning electron microscope (KYKY, Beijing, China). Regarding the TEM analysis, 20 µL of the cisplatin-loaded PBCA nanoparticles was placed on a copper grid and imaged using TEM (Zeiss, EM10C, 80 kV, Oberkochen, Germany) [62].

#### 3.3.4. Chemical Structure

To determine whether cisplatin was loaded physically or chemically into the nanoparticles, FTIR spectroscopy technique was used. Briefly, the suspension of cisplatin-loaded PBCA nanoparticles was centrifuged (20,000 RPM, 4 °C, 30 min), and the pellet was allowed to dry at room temperature. The dried pellet (2 mg) was mixed with bromide potassium (200 mg) and pressed. The obtained tablet was then analyzed using the FTIR instrument [Nicolet 740SX FTIR spectrophotometer with an MCT-B detector (Waltham, MA, USA)].

### 3.4. Drug Release Study

Drug release pattern was studied using the dialysis membrane method. For this purpose, the suspension of cisplatin-loaded PBCA nanoparticles was centrifuged (20,000 RPM, 4 °C, 30 min), and the pellet was obtained. The pellet, containing 5 mg cisplatin, was resuspended into 5 mL of fresh PBS buffer and transferred into a dialysis bag. Also, 5 mL of the standard cisplatin (1 mg/mL) was prepared in PBS and transferred to another dialysis bag. The bags were then separately immersed into 100 mL of PBS, as the acceptor medium, and stirred (150 RPM, room temperature). The water solubility of cisplatin is 1.0 mg/mL [63], confirming the maintenance of sink conditions during the experiment [17]. At the predetermined time intervals, 2 mL of PBS was replaced with 2 mL of fresh PBS, and the drug concentration in the samples was measured using the AAS method. The profile of drug release versus time was obtained using the Equation (3):

(3) $$\mathrm{Drug}\;\mathrm{release}\;(\%)=\frac{W_{\mathrm{release}}\;(\mathrm{mg})}{W_{\mathrm{total}}\;(\mathrm{mg})}\times 100$$

where W_total_ and W_release_ are the amount of the loaded drug in nanoparticles and the amount of released drug from nanoparticles into the acceptor medium at different time intervals, respectively.

### 3.5. In Vitro Cytotoxicity Effects

To evaluate the cytotoxicity effects of cisplatin and cisplatin-loaded PBCA nanoparticles, MTT assay was performed. For this purpose, ACHN and BHK-21 cells were cultured in RPMI-1640 medium supplemented with 10% FBS and 1% penicillin/streptomycin antibiotics at the density of 10^4^ cells/well in 96-well plates. After 24 h incubation (humidified incubator, 5% CO_2_, 37 °C), the cells were treated with different concentrations of cisplatin (0, 2, 4, 8, 16, 32, 64, 128, and 256 µM) in the form of standard and loaded into PBCA nanoparticles, and the plates were incubated (5% CO_2_, 37 °C). Also, the cells were incubated with a combination of free cisplatin and blank PBCA nanoparticles, as a control, at the drug concentrations mentioned above and blank nanoparticles equivalent to the cisplatin-loaded PBCA treatments. After 24, 48, and 72 h incubation, the media were removed, 100 µL of MTT (0.5 mg/mL PBS) solution was added into the wells, and the plates were incubated (5% CO_2_, 37 °C) for further 3 h. The MTT solutions were then replaced with 100 µL of isopropanol to dissolve the formazan crystals. In this assay, the positive and negative control were the cells treated with SDS + 0.1 N HCl and the cells treated with complete media, respectively. Also, only complete media was considered as the background. The absorbance was then read at 570 nm using an ELISA reader, and cell viability was calculated using the Equation (4):

(4) $$\mathrm{Cell}\;\mathrm{viability}\;(\%)=\frac{A_{\mathrm{release}}-A_{\mathrm{background}}}{A_{\mathrm{negative}\;\mathrm{control}}-A_{\mathrm{background}}}\times 100$$

In addition, the half-maximal inhibitory concentration (IC_50_) of the formulations was calculated using GraphPad PRISM software version 8.00 (GraphPad Software, Inc., San Diego, CA, USA)). All experiments were performed in triplicate.

### 3.6. In Vitro Toxicity and Therapeutic Effects

Ferric nitrilotriacetate (FeNTA) solution was prepared as a carcinogen to establish kidney cancer according to the method described previously [64] with slight modifications. Briefly, ferric nitrate and nitrilotriacetic disodium salt were separately dissolved in 120 mM sodium bicarbonate to obtain 160-mM and 320-mM solutions, respectively. Then, the solutions were mixed at the volume ratio of 1:2 (100 RPM, room temperature), and pH 7.4 was adjusted with sodium bicarbonate. The administered doses of FeNTA solution were calculated based on the Fe-content existed in the FeNTA compound.

#### 3.6.1. In Vivo Kidney Tumor Establishment

To develop kidney tumor, male Wistar rats (two weeks old, 70–80 g) were used. All animal experiments were approved by the Animal Experimental Committee of Pasteur Institute of Iran, Tehran, and all procedures were performed in accordance with the National Institute of Health Guidelines for the Care and Use of Laboratory Animals. Kidney tumor was established according to the previous study [65]. Briefly, the animals were pretreated with a single intraperitoneal injection of DEN (200 mg/kg). Fourteen days after DEN treatment, different FeNTA doses (3–9 mg FeNTA/kg) were administered intraperitoneally twice a week for 16 weeks, where the administered dose was initiated with 3 mg FeNTA/kg and gradually increased throughout the experiment, in which animals received 9 mg FeNTA/kg in 16th week.

#### 3.6.2. In Vivo Anticancer and Toxicity Effects of the Formulations

Two weeks after the last FeNTA injection, the rats were randomly divided into three groups (n = 12) and received cisplatin, cisplatin-loaded PBCA nanoparticles, and PBS as the control group. Then, cisplatin (1 mg/kg) was administered intraperitoneally at the time intervals of 72 h for three weeks. One month after the last injection, the animals were sacrificed, and tumor size was measured. To evaluate the renal function, serum creatinine and BUN were measured using creatinine and BUN measurement kits (Pars Azmoon Company, Tehran, Iran) according to manufacturer’s instructions, and the results were expressed as mg/dL. Also, the toxicity effects of the formulations (cisplatin and cisplatin-loaded PBCA nanoparticles) were histopathologically studied in kidney, liver and lung tissues using the hematoxylin and eosin (H&E) staining method.

### 3.7. Statistical Analysis

GraphPad Prism software version 8.00 was used for all statistical analyses. Statistical differences were analyzed by one-way analysis of variance (ANOVA) test. All data was expressed as mean ± standard deviation (SD, n = 3).

## 4. Conclusions

In this study, cisplatin-loaded PBCA nanoparticles were synthesized using a miniemulsion polymerization method and characterized in terms of size, size distribution, drug loading and encapsulation efficiencies, drug release behavior, in vitro cytotoxicity effects, in vivo toxicity, and therapeutic effects. This study shows that nanoparticles had a slow and controlled release of cisplatin, and for this reason, this nanoformulation was potent enough to enhance the cytotoxicity effects of cisplatin (2.3-fold) compared to the standard cisplatin. Also, the nanoformulation was sufficiently stable to inhibit the premature release of cisplatin before approaching to the target site and preserve its potency. Moreover, cisplatin-loaded PBCA nanoparticles, due to the slow and controlled drug release [66], caused a significant reduction in both BUN and creatinine concentrations compared to when using the standard cisplatin (1.6-fold and 1.5-fold, respectively). It was also demonstrated that cisplatin-loaded PBCA nanoparticles caused a 1.4-fold decrease in the mean tumor size as compared to the standard cisplatin. Overall, in vitro and in vivo results show that cisplatin-loaded PBCA nanoparticles could be a suitable drug candidate and used as a synergistic compound in association with other drugs for the treatment of kidney cancer.

## Figures and Tables

**Table 1:** Size, size distribution, and zeta potential of blank and cisplatin-loaded PBCA nanoparticles and encapsulation and loading efficiencies of cisplatin-loaded PBCA nanoparticles.

Formulations	Size (nm)	Size Distribution	Zeta Potential (mV)	Encapsulation Efficiency (%)	Loading Efficiency (%)
PBCA nanoparticles	251 ± 12.3	0.058 ± 0.002	−9 ± 0.42	-	-
Cisplatin-loaded PBCA nanoparticles	274 ± 6.7	0.046 ± 0.002	−7 ± 0.30	23	4.5

**Table 2:** Blood concentration of blood urea nitrogen (BUN) and creatinine (mg/dL) in various groups of animals, treated with different formulations (PBS, standard cisplatin, and cisplatin-loaded PBCA nanoformulation) compared to the healthy control group.

	Animal Groups	Healthy	PBS	Cisplatin	Cisplatin-Loaded PBCA
Blood Factors					
BUN (mg/dL)		16 ± 0.8	208 ± 12	172 ± 8.8	110 ± 7
Creatinine (mg/dL)		0.9 ± 0.04	12.6 ± 0.6	9 ± 0.4	6 ± 0.3
